# The Effect of Chronic Kidney Disease on a Physical Activity Intervention: Impact on Physical Function, Adherence, and Safety

**DOI:** 10.23937/2572-3286.1510021

**Published:** 2017-02-14

**Authors:** CK Liu, J Milton, F-C Hsu, KM Beavers, V Yank, T Church, JD Shegog, S Kashaf, S Nayfield, A Newman, RS Stafford, B Nicklas, DE Weiner, RA Fielding, LIFE-P Research Group

**Affiliations:** 1 Nutrition, Exercise Physiology, and Sarcopenia Laboratory, Jean Mayer Human Nutrition Research Center in Aging, Tufts University, Boston, MA, USA; 2 Boston University School of Medicine, Boston, MA, USA; 3 Boston University School of Public Health, Boston, MA, USA; 4 Wake Forest School of Medicine, Winston-Salem, NC, USA; 5 Stanford University School of Medicine, Palo Alto, CA, USA; 6 Pennington Biomedical Research Center, Baton Rouge, LA, USA; 7 Yale University School of Medicine, New Haven, CT, USA; 8 University of Florida College of Medicine, Gainesville, FL, USA; 9 University of Pittsburgh School of Public Health, Pittsburgh, PA, USA; 10 Tufts University School of Medicine, Boston, MA, USA

**Keywords:** CKD, Physical activity, Older adults, SPPB

## Abstract

**Background:**

Because chronic kidney disease (CKD) is associated with muscle wasting, older adults with CKD are likely to have physical function deficits. Physical activity can improve these deficits, but whether CKD attenuates the benefits is unknown. Our objective was to determine if CKD modified the effect of a physical activity intervention in older adults.

**Methods:**

This is an exploratory analysis of the LIFE-P study, which compared a 12-month physical activity program (PA) to a successful aging education program (SA) in older adults. CKD was defined as a baseline eGFR < 60 mL/min/1.73 m^2^. We examined the Short Physical Performance Battery (SPPB) at baseline, 6 and 12 months. Secondary outcomes included serious adverse events (SAE) and adherence to intervention frequency. Linear mixed models were adjusted for age, sex, diabetes, hypertension, CKD, intervention, site, visit, baseline SPPB, and interactions of intervention and visit and of intervention, visit, and baseline CKD.

**Results:**

The sample included 368 participants. CKD was present in 105 (28.5%) participants with a mean eGFR of 49.2 ± 8.1 mL/min/1.73 m^2^. Mean SPPB was 7.38 ± 1.41 in CKD participants; 7.59 ± 1.44 in those without CKD (p = 0.20). For CKD participants in PA, 12-month SPPBs increased to 8.90 (95% CI 8.32, 9.47), while PA participants without CKD increased to 8.40 (95% CI 8.01, 8.79, p = 0.43). For CKD participants in SA, 12-month SPPBs increased to 7.67 (95% CI 7.07, 8.27), while participants without CKD increased to 8.12 (95% CI 7.72, 8.52, p = 0.86). Interaction between CKD and intervention was non-significant (p = 0.88). Number and type of SAEs were not different between CKD and non-CKD participants (all p > 0.05). In PA, adherence for CKD participants was 65.5 ± 25.4%, while for those without CKD was 74.0 ± 22.2% (p = 0.12).

**Conclusion:**

Despite lower adherence, older adults with CKD likely derive clinically meaningful benefits from physical activity with no apparent impact on safety, compared to those without CKD.

## Introduction

Older adults with chronic kidney disease (CKD) are vulnerable to disability [1,2]; CKD doubles the odds that an older person will develop difficulties in their activities of daily living [3]. Several factors likely contribute to this phenomenon. CKD complications include anemia [4], vitamin D deficiency [5], and cardiovascular disease [6], all of which can adversely affect the musculoskeletal system. Older age is associated with sarcopenia, a condition of reduced muscle mass and quality. On a molecular level, the myostatin protein, which represses skeletal muscle growth, is upregulated in age [7] and CKD [8]. Prior studies demonstrated that muscle atrophy increases as kidney function decreases [9].

Physical activity improves physical function in other populations of older adults with chronic conditions [10]; prior studies of physical activity in older adults with CKD have been fairly modest in sample size [11–18]. Taking data from the Lifestyle Interventions and Independence for Elders Pilot (LIFE-P) Study, a 12-month randomized controlled trial of supervised physical activity in over 400 older adults, we performed a post-hoc analysis to examine if CKD affected potential changes in physical function. For our primary outcome, we compared changes in Short Physical Performance Battery (SPPB) scores, which measures general physical function. For secondary outcomes, we examined the occurrence of serious adverse events and adherence to the intervention, as both will be key for successful implementation of physical activity interventions in individuals with CKD.

## Materials and Methods

### Study design

The LIFE-P study was a single-blind multi-center randomized controlled trial (Cooper Institute, Dallas, TX; Stanford University, Palo Alto, CA; University of Pittsburgh, Pittsburgh, PA; and Wake Forest University, Winston-Salem, NC), comparing the efficacy of a 12-month physical activity intervention (PA) to a successful aging health education program (SA) on the incidence of mobility disability in functionally limited older adults [19]. The study design and results of LIFE-P have previously been published [20]. Institutional Review Boards of all sites approved the study and all participants provided written informed consent.

### Study participants

At baseline, participants were 70 to 89 years old, able to walk 400 meters unassisted in ≤ 15 minutes [21], sedentary, and scored ≤ 9 on the SPPB [20]. A total of 424 participants were followed for 12 to 18 months. To be eligible for this analysis, participants were required to have baseline data on serum creatinine and the SPPB.

### Intervention

Participants randomized to PA underwent aerobic, strength, balance and flexibility exercises for 12–18 months, with walking as the primary activity. For the first two months, sessions occurred thrice weekly and were supervised at the study site. During months three to six, supervised sessions occurred twice weekly and home-based exercises were initiated. At month seven, participants were transitioned to a home-based program with an optional weekly center-based session. After month six, participants were periodically contacted by phone to encourage adherence. Participants were instructed to perform activity at a moderate level defined by the Borg scale [22].

Participants randomized to SA participated in workshops about relevant health topics, such as nutrition and advanced care planning. Sessions occurred weekly during months one through six, then monthly starting at month seven. After a missed session, participants were contacted by phone to encourage attendance. At the conclusion of each session, participants did five to ten minutes of gentle upper extremity stretching.

### Participant characteristics

Height, weight, sex, and race were recorded. Body mass index (BMI) was calculated from height and weight. History of diabetes, hypertension, cardiovascular disease (stroke, myocardial infarction, and congestive heart failure), or chronic pulmonary disease was self-reported and collected at baseline. A full description of the baseline blood draw has been previously published [23]. Baseline creatinine was measured using an Integrated Database Management System calibrated assay and eGFR was calculated using the CKD-EPI equation [24]. CKD was defined as a baseline eGFR < 60 mL/min/1.73 m^2^ [25].

### Short Physical Performance Battery (SPPB)

The SPPB is a composite measure of physical functioning that assesses gait speed, lower extremity strength, and balance. For gait speed, participants walked four meters at their usual pace. For lower extremity strength, participants stood up from a seated position five times in a row without stopping or using arms. For balance, participants attempted three standing positions: (1) feet side by side, (2) heel of foot beside toe of other foot, and (3) heel of foot in front of the other foot. Participants scored between zero and four on each task; the sum comprised the SPPB score (range 0–12) [26].

### Adherence to intervention frequency

The number of sessions attended was compared to the number of sessions available for each phase and for study duration, excluding closings (e.g. holidays or inclement weather) and leaves for medical reasons (e.g. surgery or hospitalization).

### Serious adverse events

Any deaths, life threatening events, inpatient hospitalizations, or clinically significant laboratory or diagnostic test abnormalities that required immediate medical attention was documented. Such information was collected during assessments, intervention sessions, or reminder calls. All events, study-related or not, were included in the analysis.

### Statistical analyses

Baseline demographics and physical performance measures by CKD status were compared using two sample t-tests for continuous variables and the chi-square test for categorical variables. Using a linear mixed model, SPPB scores by CKD status and intervention were compared, adjusting for age, sex, hypertension, diabetes, study site, baseline SPPB score, and the interactions of intervention with visit and of intervention, visits and baseline CKD. Within this model, we also checked for the interactions of: 1) baseline CKD and intervention, and 2) baseline CKD and visit. A subgroup analysis of participants with baseline CKD was also performed, adjusting for age, sex, hypertension, diabetes, study site, visit, baseline SPPB score, and interaction of intervention and visit. Least squares means for SPPB scores with 95% CI are presented.

The number of serious adverse events at twelve months by CKD status and intervention group was compared with the chi-square test. Mean rates of adherence were compared using the Wilcoxon rank sum test. Data are expressed as mean values with standard deviation. Analyses were performed using SAS (version 9.3, SAS Institute, Inc., Cary, NC).

## Results

### Baseline characteristics

**Figure 1:**
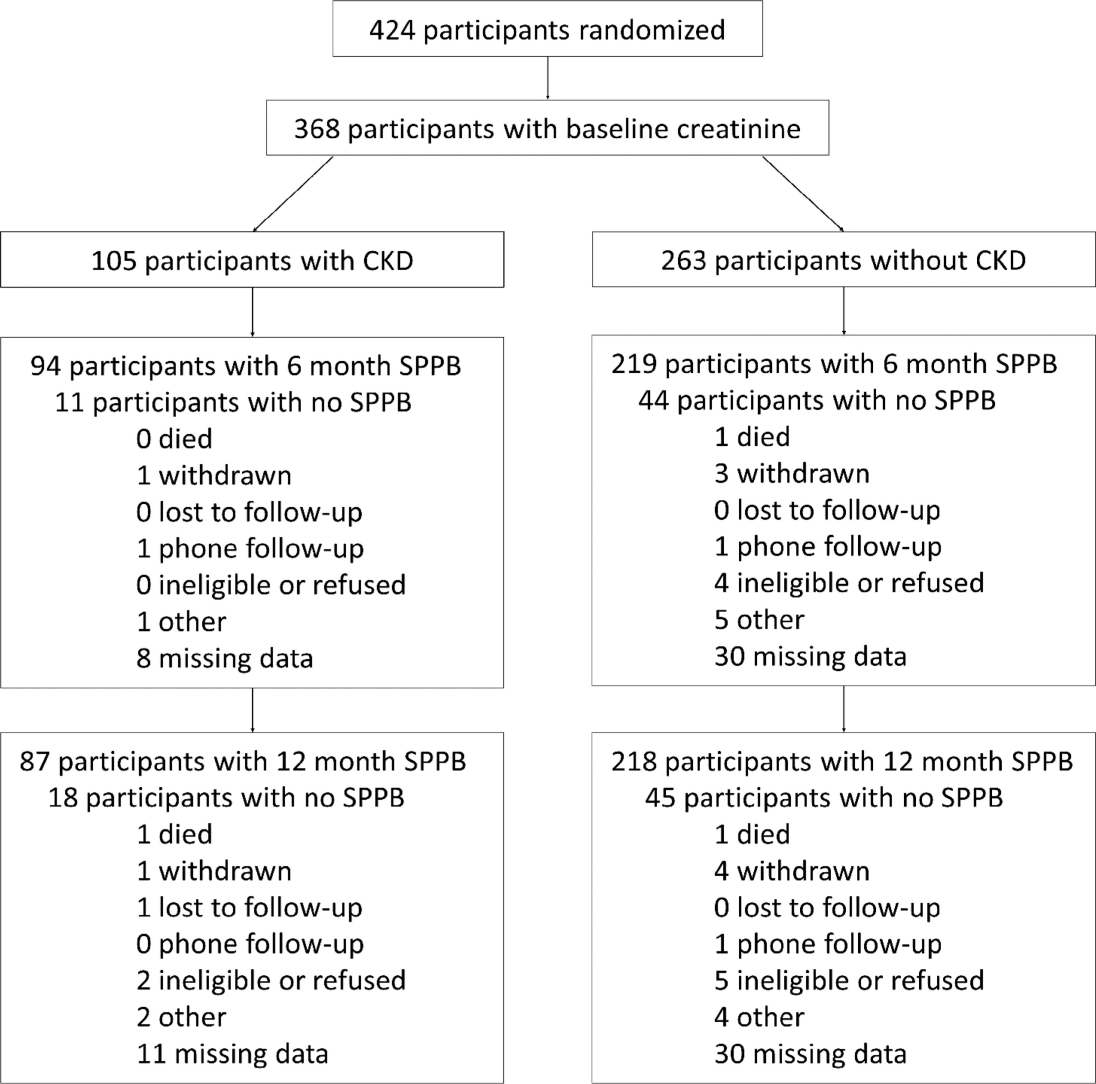
Flow of participants into sample.

A total of 424 participants were enrolled into the study (Figure 1). All participants consented to the baseline blood draw, with adequate sample obtained from 368 participants (87%). At six months, 313 participants returned for a follow-up; at twelve months, 295 participants returned. Of the original 105 participants with CKD, 87 (83.9%) had a 12-month follow-up, while 218 (82.9%) of the original 263 non-CKD participants underwent follow-up at twelve months. Of those with baseline CKD, 27 (25.7%) had an eGFR ≤ 45 ml/min/m^2^, or stage IIIB CKD. Equivalent proportions of CKD and non-CKD participants were randomized to PA. Participants with CKD were older and more likely to have diabetes and hypertension at baseline. No difference was found in baseline SPPB scores; those with CKD had a baseline SPPB score of 7.38 ± 1.41, while those without CKD had a score of 7.59 ± 1.44 (p = 0.20, Table 1).

### Impact of physical activity on short physical performance battery

At 12 months, all participants randomized to PA had greater mean SPPB scores compared to their SA counterparts (Table 2) regardless of baseline CKD. PA participants with CKD improved their SPPB scores by 1.27 points from baseline, while PA participants without CKD increased by 0.97 points (p = 0.43). For those randomized to SA, CKD did not affect the follow-up mean SPPB scores at either six (p = 0.87) or twelve months (p = 0.86). Subgroup analysis of those with CKD alone determined that the intervention resulted in statistically significant differences in SPPB scores (overall p = 0.02, Table 3). None of the interactions checked were statistically significant, including the interaction of intervention with CKD (p = 0.88) and of visit, and baseline CKD (p = 0.27).

### Participant adherence to intervention frequency

**Figure 2:**
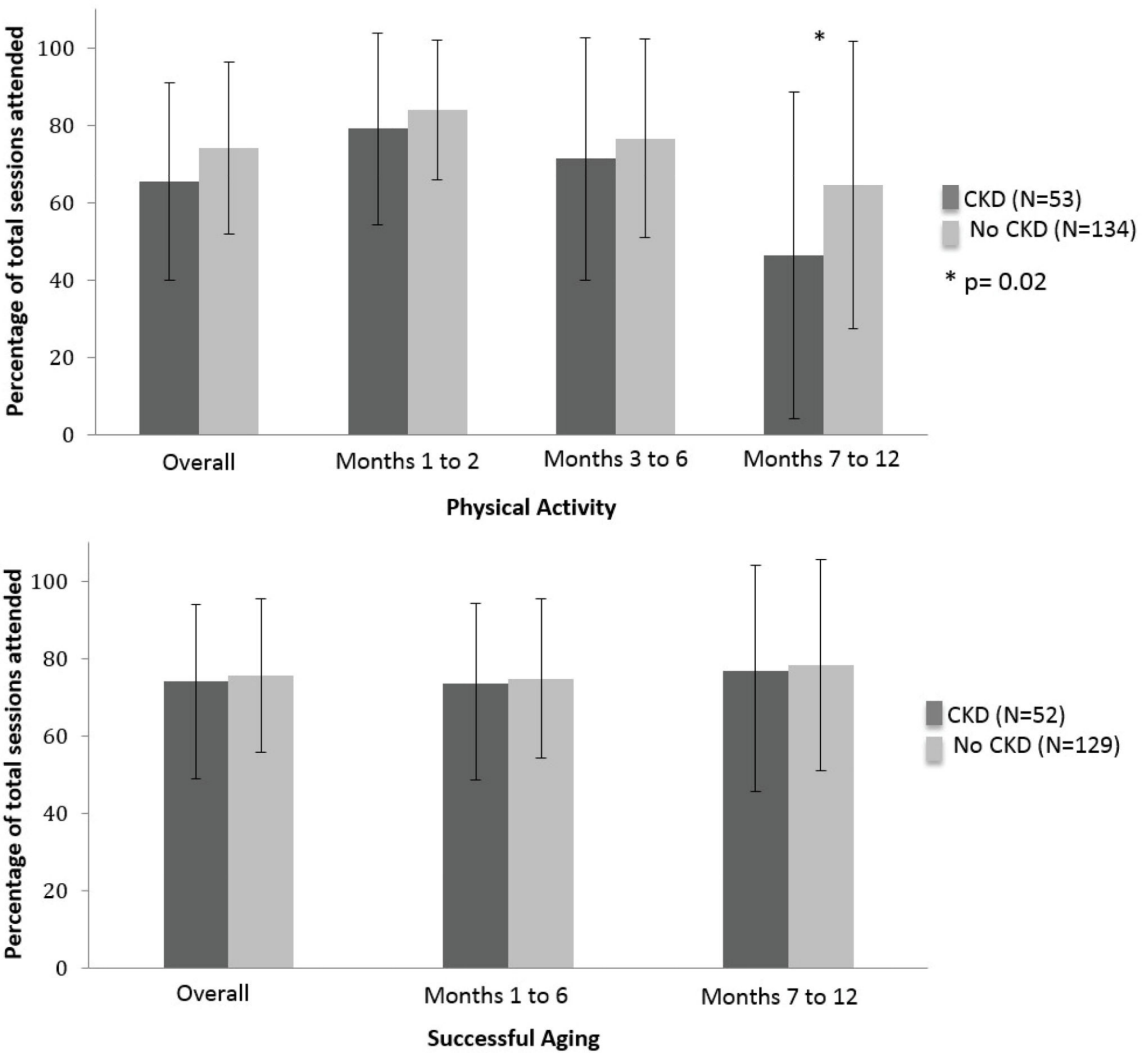
Adherence to intervention frequency by baseline CKD status^a^. ^a^Sessions attended compared to sessions available. Bars represent standard deviations; Physical activity: months 1 to 2: three center-based sessions/week; months 3 to 6: two center-based and one home-based session/week; months 6 to 12: one optional center-based and/or three home-based sessions/week; Successful aging: months 1 to 6: one session/week; months 7 to 12: one session/month. For PA, p = 0.12 for overall adherence, p = 0.45 for months 1 and 2, p = 0.39 for months 3 to 6, and p = 0.02 for months 7 to 12. For SA, p = 0.60 for overall adherence, p = 0.56 for months 1 to 6, and p = 0.42 for months 7 to 12.

PA participants with CKD had an overall adherence rate of 65.5 ± 25.4% (Figure 2), while the rate in those without CKD was 74.0 ± 22.2% (p = 0.12). In all PA study phases, individuals with CKD had lower adherence than those without CKD. However, these results only achieved statistical significance in the last six months of the study. During this phase, CKD participants had an attendance rate of 46.4 ± 42.1%, while the attendance rate for those without CKD was 64.5 ± 37.1%. In the SA group, adherence did not differ by CKD status during any phases of the study. The overall attendance rates for SA were 74.3 ± 18.7% for participants with CKD versus 75.7 ± 19.8% for those without CKD (p = 0.56).

### Adverse events

Three deaths occurred in the sample; there were no episodes of severe disability. Overall, there were very few life threatening events (Table 4). For PA and SA, the proportion of inpatient hospitalizations was numerically higher in those with baseline CKD compared to those without CKD, but the differences in proportions was not statistically significant. Similarly, there were no statistical significant differences between groups based on CKD status and intervention arm for the event of clinically significant laboratory or diagnostic findings (all p > 0.05).

## Discussion

In this exploratory study, we found that a 12-month program of physical activity improved SPPB scores. In older adults with CKD, the improvements were no different when compared to the changes seen in older adults with normal kidney function. Notably, this was in the context of somewhat lower rates of intervention adherence among the CKD participants. During the study, there were no differences in the number of adverse events occurring in CKD and non-CKD participants.

The study showed that physical activity improved SPPB scores with CKD, an overall measure of physical function in older adults [26]. SPPB scores increased by 1.27 points, a clinically meaningful change; the minimum clinical important difference for the SPPB is 0.5 points [27]. Our results complement prior research on functional performance in CKD patients. In a study of 29 men with Stage 3 to 4 CKD, 12 weeks of physical activity improved sit to stand times, compared to a control group [12]. Similarly, another study of 16 older adults with Stage 4 CKD demonstrated that 12 weeks of physical activity also improved performance on the ‘Get Up and Go’ test [13]. Six months of physical activity improved performance on the 30 second ‘Sit-to-Stand’ test in 47 persons with CKD [14]. Four studies demonstrated that a physical activity intervention of 12 or more weeks improved performance on the six meter walk test in CKD patients [12–15].

Our study adds to the literature in several ways. First, CKD is highly prevalent in older adults, but the majority of prior studies regarding physical activity in CKD have not focused on this population. Only older persons were eligible, making our study more reflective of the general patient population. Second, compared to the prior studies on physical activity in persons with CKD, our study was larger in size and had a diverse study population with over 18% African-Americans. Inclusion of African-Americans is important, as they are more likely to have severe CKD [28] and are more vulnerable to losses in physical function [29]. Finally, the intervention was home-based during the second half of the study. The majority of previous studies utilized a physical activity intervention that was highly supervised and usually required attendance at the study site. Our results therefore suggest that less supervised forms of physical activity may still be beneficial in those with CKD.

There were no statistically significant differences in CKD status for serious adverse events. A study with 72 CKD patients testing a 12-month program of physical activity found similar findings [15]. In the United Kingdom, a study of CKD, dialysis, and kidney transplant patients also found that physical activity did not impact the number of adverse events [16]. To date, 11 trials of physical activity have been conducted in CKD patients; none have reported a notable increase in serious adverse events with the interventions groups. Coupled with our data, the evidence suggests that physical activity is likely safe for older adults with CKD.

Regarding adherence, this is one of the largest studies to focus on this aspect of physical activity. Two modest studies of center-based physical activity in CKD patients found overall attendance rates of 80 to 84% [17,18] for a 12-month intervention, while another study of 72 patients demonstrated a mean attendance rate of 70% for eight weeks of center-based physical activity [15]. Preliminary data from an ongoing clinical trial of physical activity in older adults with CKD found intervention adherence was 76% [30]. In our study, attendance rates were consistently lower in CKD patients and statistically significant for CKD participants in PA during the last six months of the study. This finding of lower attendance for CKD participants suggests that the reduced adherence may be linked to CKD itself. Although there has been little research comparing study adherence rates of those with and without CKD, one possible reason for the disparity may be the increased co-morbidity burden of those with CKD, translating into a higher risk of developing illness [31]. Poor kidney function is often associated with fatigue and reduced endurance. A qualitative study found CKD patients often experienced exhaustion [32], and others have shown that scores on the vitality/ energy domain of the Short Form-36 decreased as eGFR declined [33]. Moreover, the difference in attendance rates was greatest during the last six months of the study when intervention transitioned to a home-based program and the in-center sessions became optional. A fatigued person is less likely to attend an optional session. Of note, attendance dropped for participants with CKD and without CKD, suggesting there are other factors not related to CKD that underlie attendance. This global decrease in attendance during the last half of the study likely impacted the 12-month follow-up SPPB scores, which were lower than the 6-month follow-up SPPB scores for both participants with CKD and without CKD.

The results from this exploratory study suggest that physical activity is likely safe and beneficial for older adults with CKD. This occurred despite lower adherence rates and potential catabolic effects of CKD on muscle, suggesting that skeletal muscle in older adults remains resilient. Clinically, older adults with CKD should be encouraged to undertake physical activity despite their greater comorbidity burden. In addition to the potential benefits on function, endurance and strength, physical activity can reduce the likelihood of mobility problems [10], disability, and even mortality. In a study of patients with end stage renal disease, each additional ten minutes of physical activity a day was associated with a 22% reduction in risk of death [34]. Moreover, regular physical activity is linked with quality of life; one study demonstrated that three months of physical activity improved energy levels and sleep quality as measured by the Kidney Disease Quality of Life Short Form [35].

There are several strengths of this work. First, this is the largest study to date of examining the impact of physical activity in older adults with CKD. Second, the intervention was for 12 months while many other studies of physical activity in CKD patients have been for shorter duration. Third, our study specifically examined adherence and the safety of the physical activity intervention, which is needed to determine the feasibility of physical activity on a large scale for older adults with CKD.

Several limitations should be noted. The study was exploratory in nature and a post-hoc analysis; thus, this study had limited power to detect changes in our outcome of interest. Regarding the modest sample size, the LIFE-P cohort was a pilot study for a larger trial, which has now completed [10]. However, we chose not to utilize the data of the larger trial as it was longer in duration (mean follow-up time 2.6 years versus 1.2 years for our study), likely magnifying the impact of competing risks. Most of the CKD participants had Stage 3 or moderate CKD while only a modest fraction of the sample (25.7%) had Stage 4 or severe CKD. As eGFR worsens, physical function declines [36], likely in part due to the increased degree of skeletal muscle catabolism from uremia [9]. Therefore, an intervention that is effective for an older person with Stage 3 may not be effective for a person with stage 5 CKD. Those with worse CKD may require a physical activity intervention that is different in amount or intensity to compensate for the uremia-related muscle loss. We lacked measurements of albuminuria and cystatin C, which improve the kidney function assessment. Cystatin C has been particularly shown to improve eGFR assessment in older adults, especially in those with low muscle mass [37]. However, as study eligibility required participants to have some physical limitations at baseline, low muscle mass was likely in these individuals, suggesting the severity of CKD in our sample may have actually been greater than we were able to assess.

## Conclusions

Twelve months of physical activity resulted in physical function improvements compared to a successful aging educational program in older adults with CKD; the effects were no different from those observed in older adults with normal kidney function. Although our study was exploratory, the results suggest that physical activity is likely an effective and safe modality for this population, and older adults with CKD should be encouraged to undertake regular physical activity. Next steps will focus on determining whether the benefits of physical activity persist over time and confirming our results using data from the subsequent full LIFE trial. Future directions will explore ways to encourage and implement programs of regular physical activity in older adults with CKD to improve function and quality of life.

## Supplementary Material

Supplemental file

## Figures and Tables

**Table 1:** Characteristics of study sample at baseline^a^.

Characteristic	All participants (*n* = 368)	CKD (*n* = 105)	No CKD (*n* = 263)	P-value
Age (years)	76.7 ± 4.3	77.7 ± 3.9	76.3 ± 4.3	< 0.01
Women (%)	251 (68.2)	69 (65.7)	182 (69.2)	0.53
White (%)	286 (77.7)	82 (78.1)	204 (77.6)	1.00
African American (%)	69 (18.8)	23 (21.99)	46 (17.5)	0.33
Physical activity intervention (%)	182 (49.5)	53 (50.5)	129 (49.1)	0.82
Current smokers (%)	53 (14.4)	18 (17.1)	33 (13.3)	0.88
Body mass index (kg/m^2^)	30.2 ± 5.8	30.7 ± 5.5	30.0 ± 5.9	0.29
Diabetes mellitus (%)	84 (22.8)	38 (36.2)	46 (17.5)	< 0.01
Hypertension (%)	255 (69.5)	83 (79.1)	172(65.7)	0.01
Cardiovascular disease (%)^b^	50 (13.6)	19 (18.1)	31 (11.8)	0.13
Serum creatinine (mg/dL)	0.94 ± 0.28	1.24 ± 0.27	0.82 ± 0.16	< 0.01
Estimated glomerular filtration rate (ml/min/1.73 m^2^)	69.6 ± 16.8	49.2 ± 8.1	77.66 ± 11.8	< 0.01
Short physical performance battery score, points	7.53 ± 1.43	7.38 ± 1.41	7.59 ± 1.44	0.20

a All characteristics are mean ± SD or N (%);

b Cardiovascular disease: stroke, myocardial infarction, and/or congestive heart failure;

CKD: Chronic Kidney Disease; SD: Standard Deviation.

**Table 2:** Effect of physical activity in participants with and without baseline CKD on least square means Short Physical Performance Battery scores^a^.

	CKD (95% CI)	No CKD (95% CI)	P-value
	N = 105	N = 263	
PA			
Baseline	7.62 (7.26,7.99)	7.60 (7.33,7.86)	
6 months	9.02 (8.51,9.54)	8.76 (8.40,9.13)	0.86
12 months	8.90 (8.32,9.47)	8.40 (8.01,8.79)	0.43
SA			
Baseline	7.13 (6.73,7.54)	7.59 (7.36,7.82)	
6 months	7.85 (7.85,8.39)	8.12 (7.75,8.49)	0.87
12 months	7.67 (7.07,8.27)	8.82 (7.72,8.52)	0.86

a Adjusted for age, sex, diabetes, hypertension, CKD, intervention, site, visit, baseline SPPB score, and interaction of intervention and visit; P value for interaction of intervention with visit = 0.42;

CKD: Chronic Kidney Disease; CI: Confidence Interval; PA: Physical Activity; SA: Successful Aging.

**Table 3:** Least squares mean Short Physical Performance Battery (SPPB) scores in points at 6 and 12 months only in persons with baseline CKD^a^.

	SPPB scores (95% CI)		
Intervention	Baseline	6 months	12 months
Physical activity (N = 53)	7.65 (7.28,8.02)	8.44 (7.92,8.95)	8.25 (7.68,8.82)
Successful aging (N = 52)	7.19 (6.77,7.61)	7.73 (7.15,8.31)	7.43 (6.78,8.10)
P value	0.10	0.04	0.04
Overall p value	0.02		

a Adjusted for age, sex, diabetes, hypertension, site, visit, baseline SPPB score, and interaction of intervention and visit; P value for interaction of intervention with visit = 0.73;

CKD: Chronic Kidney Disease; CI: Confidence Interval; PA: Physical Activity; SA: Successful Aging.

**Table 4:** Number (%) of serious adverse events in those with (N = 105) and without (N = 263) baseline CKD.

Adverse event	CKD status	PA	SA	P-value

Death	CKD	0 (0%)	1(2%)	0.33

	No CKD	2 (2%)	0 (0%)	

Life-threatening events	CKD	1 (2%)	1 (2%)	1.0

	No CKD	3 (2%)	2 (1%)	

Inpatient hospitalizations	CKD	17 (32%)	17 (33%)	0.73

	No CKD	29 (22%)	25 (19%)	

Clinically significant laboratory or diagnostic abnormalities^a^	CKD	5 (9%)	3 (6%)	0.58

	No CKD	10 (8%)	10 (7%)	

a Must have required immediate medical attention;

CKD: Chronic Kidney Disease; PA: Physical Activity; SA: Successful Aging.
