# Supplementary material for: The Effect of Chronic Kidney Disease on a Physical Activity Intervention: Impact on Physical Function, Adherence, and Safety
Source: J Clin Nephrol Ren Care. Author manuscript; Available in PMC 2018 May 7. (PMC5937279; doi:10.23937/2572-3286.1510021)
Supplement: Supplemental file [file NIHMS923429-supplement-Supplemental_file.doc]

**Appendix: Research Investigators for LIFE Pilot study**

**Cooper Institute, Dallas, TX:**

Steven N. Blair, P.E.D. – Field Center Principal Investigator

Timothy Church, M.D., Ph.D., M.P.H. – Field Center Co-Principal Investigator

Jamile A. Ashmore, Ph.D.

Judy Dubreuil, M.S.

Georita Frierson, Ph.D.

Alexander N. Jordan, M.S.

Gina Morss, M.A.

Ruben Q. Rodarte, M.S.

Jason M. Wallace, M.P.H.

**National Institute on Aging**

Jack M. Guralnik, M.D., Ph.D. – Co-Principal Investigator of the Study

Evan C. Hadley, M.D.

Sergei Romashkan, M.D., Ph.D.

**Stanford University, Palo Alto, CA**

Abby C. King, Ph.D. – Field Center Principal Investigator

William L. Haskell, Ph.D. – Field Center Co-Principal Investigator

Leslie A. Pruitt, Ph.D.

Kari Abbott-Pilolla, M.S.

Karen Bolen, M.S.

Stephen Fortmann, M.D.

Ami Laws, M.D.

Carolyn Prosak, R.D.

Kristin Wallace, M.P.H.

**Tufts University**

Roger Fielding, Ph.D.

Miriam Nelson, Ph.D.

Dr. Fielding's contribution is partially supported by the U.S. Department of Agriculture, under agreement No. 58-1950-4-401. Any opinions, findings, conclusion, or recommendations expressed in this publication are those of the author(s) and do not necessarily reflect the view of the U.S. Dept of Agriculture.

**University of California, Los Angeles, Los Angeles, CA**

Robert M. Kaplan, Ph.D., M.A.

**VA San Diego Healthcare System and University of California, San Diego, San Diego, CA**

Erik J. Groessl, Ph.D.

**University of Florida, Gainesville, FL**

Marco Pahor, M.D. – Principal Investigator of the Study

Michael Perri, Ph.D.

Connie Caudle

Lauren Crump, M.P.H

Sarah Hayden

Latonia Holmes

Cinzia Maraldi, M.D.

Crystal Quirin

**University of Pittsburgh, Pittsburgh, PA**

Anne B. Newman, M.D., M.P.H. – Field Center Principal Investigator

Stephanie Studenski, M.D., M.P.H. – Field Center Co-Principal Investigator

Bret H. Goodpaster, Ph.D., M.S.

Nancy W. Glynn, Ph.D.

Erin K. Aiken, B.S.

Steve Anthony, M.S.

Judith Kadosh, B.S.N., R.N.

Piera Kost, B.A.

Mark Newman, M.S.

Christopher A. Taylor, B.S.

Pam Vincent, C.M.A.

Diane Ives, M.P.H

The Pittsburgh Field Center was partially supported by the Pittsburgh Claude D. Pepper Center P30 AG024827.

**Wake Forest University, Winston-Salem, NC**

Stephen B. Kritchevsky, Ph.D. – Field Center Principal Investigator

Peter Brubaker, Ph.D.

Jamehl Demons, M.D.

Curt Furberg, M.D., Ph.D.

Jeffrey A. Katula, Ph.D., M.A.

Anthony Marsh, Ph.D.

Barbara J. Nicklas, Ph.D.

Jeff D. Williamson, M.D., M.P.H.

Rose Fries, L.P.M.

Kimberly Kennedy

Karin M. Murphy, B.S., M.T. (ASCP)

Shruti Nagaria, M.S.

Katie Wickley-Krupel, M.S.

*Data Management, Analysis and Quality Control Center (DMAQC)*

Michael E. Miller, Ph.D. – DMAQC Principal Investigator

Mark Espeland, Ph.D. – DMAQC Co-Principal Investigator

Fang-Chi Hsu, Ph.D.

Walter J. Rejeski, Ph.D.

Don P. Babcock, Jr., P.E.

Lorraine Costanza

Lea N. Harvin

Lisa Kaltenbach, M.S.

Wei Lang, Ph.D.

Wesley A. Roberson

Julia Rushing, M.S.

Scott Rushing

Michael P. Walkup, M.S.

The Wake Forest University Field Center is, in part, supported by the Claude D. Older American Independence Pepper Center #1 P30 AG21332.

**Yale University**

Thomas M. Gill, M.D.

Dr. Gill is the recipient of a Midcareer Investigator Award in Patient-Oriented Research (K24AG021507) from the National Institute on Aging.
